# The effect of parity on time to initiate complementary feeding among mother-infant pairs in Awi Zone, Northwest Ethiopia

**DOI:** 10.1186/s13052-024-01612-1

**Published:** 2024-03-13

**Authors:** Tilahun Degu Tsega, Gashaw Melkie Bayeh, Kassaye Demeke Alemu, Abaynew Assemu Asrade, Abebaw Molla Kebede, Tamiru Alene, Zewdu Bishaw Aynalem, Bezawit Adane, Melaku Yalew, Molla Getie Mehari, Almaw Genet Yeshiwas, Tadesse Miretie Dessie, Eniyew Talie Fenta, Kefale Mitiku Haylu

**Affiliations:** 1 Department of Public Health, College of Medicine and Health sciences, Injibara University, Injibara, Ethiopia; 2 Department of HIV/AIDS Prevention, Care and treatment, Amhara regional health bureau, Bahir Dar, Ethiopia; 3 Department of Pediatric Nursing, College of Medicine and Health sciences, Injibara University, Injibara, Ethiopia; 4 Department of Nursing, College of Medicine and Health sciences, Injibara University, Injibara, Ethiopia; 5 Department of Medical Laboratory Sciences, College of Medicine and Health Sciences, Injibara University, Injibara, Ethiopia; 6 Department of Environmental health, College of medicine and Health Sciences, Injibara University, Injibara, Ethiopia; 7 Deparment of Biomedical Sciences, College of Medicine and Health Sciences, Injibara University, Injibara, Ethiopia

**Keywords:** Complementary feeding, Parity, Effect, Mothers, Infants, Median time

## Abstract

**Introduction:**

Despite strategies and recommendations for complementary feeding initiation were applied globally, mothers initiated complementary feeding to the infants on time was low. Previous works of literatures were not identified the effect of parity on time to initiate complementary feeding. Particularly, evidences regarding to this in Ethiopia is scanty. Therefore, this study aimed to identify the effect of parity on time to initiate complementary feeding among mother-infants pairs in Northwest Ethiopia.

**Methods:**

A community-based prospective cohort study was carried out among 732 primipara, and 1464 multipara mothers who had a live birth in Northwest Ethiopia. Data were collected using Kobo collect software at the start of and on a monthly bases until the end of the follow up period. Parity as exposure variable and other confounders were analyzed using cox proportional hazard regression. Kaplan-Meier survival curve and the Schoenfeld residuals global test (P-value = 0.4861) was performed. Hazard ratio (HR) with 95% confidence intervals (CI) was used to declare statistical significance of predictors.

**Results:**

The overall incidence rate of initiation of complementary feeding among primipara and multipara mothers were 16.27 (95%CI: 15.04, 17.61) and 13.30 (95%CI: 12.53, 14.12) person months’ observations respectively. The median time to initiate complementary feeding among primipara and multipara mothers for their infants was 5 and 6 months respectively. Primipara mothers had a 30% higher rate to initiate complementary feeding early (AHR = 1.30, 95%CI: 1.17, 1.43). Age from 15 to 24 and 25–34 years (AHR = 1.69, 95%CI: 1.36, 2.09; and AHR = 1.45, 95%CI: 1.17, 1.81) and Birth type (twin) (AHR = 1.29, 95%CI: 1.02, 1.64) were statistically significant predictors for time to initiate complementary feeding.

**Conclusions:**

Parity was identified as a statistically significant predictor for time to initiate complementary feeding. The incidence rate of early and late initiation of complementary feeding was higher among primipara than multipara mothers. Besides, the median time to initiate complementary feeding was earlier among primipara than multipara mothers. So, a parity based complementary feeding practice education should be advocated to tackle the gap and further reduce infants and children malnutrition. Relatively younger age and twin delivered mothers initiated complementary feeding against the recommendation. Therefore, intervention considering such statistically significant predictors could have a public health importance.

**Supplementary Information:**

The online version contains supplementary material available at 10.1186/s13052-024-01612-1.

## Introduction

In addition to breastfeeding, children can be given complementary foods, which are solid, semi-solid, and silky foods made either commercially or at home [1]. Infants and children between the ages of six and twenty-four months should get nutritionally adequate meals in addition to breast milk [2–4]. The infant’s first meaningful proactive development step is the introduction of complementary nutrition. Following a series of neurodevelopmental achievements complementary feeding turns into a socialization tool [2, 3]. Stunting, wasting, micronutrient deficiencies, overweight, obesity, and diet-related non-communicable diseases can all be avoided during the complementary feeding phase [5].

The World Health Organization (WHO) and the United Nations Children’s Fund (UNICEF) advised that infants receive complementary feedings starting at 6 months of age and continuing for at least 24 months [1, 3]. UNICEF also took into account the fact that children who are fed enough nutrient-dense foods in the proper proportions at the right stages of development had a higher chance of surviving, growing, developing, and learning. They are better positioned to thrive even in the face of illness, disaster, or crisis [1]. Three Sustainable Development Goals (SDGs)—improving nutrition (SDG-2), lowering child mortality and the risk of non-communicable diseases (SDG-3), and enhancing cognitive development and education (SDG-4)—can only be achieved with the help of complementary feeding [6].

Breast milk supplemented with meals before the age of six months is unnecessary and discouraged due to the risk of contamination and the increased risk of infectious diseases. Because the frequency and intensity of suckling increase the production and release of breast milk, starting additional feeding too soon reduces breast milk output. Breast milk is no longer sufficient to cover the infant’s nutritional demands after six months, so other foods should be added to the child’s diet [1–3, 7, 8].. Implementing complementary feeding methods on time can help children live, grow, and develop, as well as prevent micronutrient deficiencies, morbidity, and obesity later in life [1, 8, 9]. Even though proper complementary feeding practice could save millions lives every year [10–13], the prevalence of undernutrition among children aged 6 to 24 is high due to the introduction of breastmilk substitutes either too early or too late and negatively impact their growth as well as development [14, 15].

The number of live births after at least 20 weeks of pregnancy is the most frequently accepted definition of parity. Multiparity is defined as having many births following fetal viability, as opposed to primiparity, which is defined as having only one birth [16]. In Ethiopia, primiparity is defined as having only one child after and at 28 weeks of gestation [17].

Worldwide, the percentage of mothers of all parity who started complementary feeding on time was low, and the timely starting of feeding was below recommended levels [1, 18, 19]. Around 45% of child mortalities in the middle of 2021 were attributable to malnutrition; in the same manner, over 144 million children under the age of five are underweight for their age worldwide. Furthermore, it was stated that 45 and 38.9 million under-five children globally were obese and wasting, respectively, by 2020. This number is significantly greater in developing nations where childhood obesity and overweight are on the rise simultaneously [19]. Ethiopia continues to have high rates of wasting and stunting along with significant newborn and infant mortality [7, 18]. This could be related to the low percentage of complementary feedings that are started on time. Additionally, the percentage varies between nations based on regional factors like wealth, food, and cultural differences [20–24].

Malnutrition has a severe and long-lasting effect on social, economic, health, and developmental issues for individuals, families, communities, and the nation as a whole. One of the most difficult public health problems in the world is the fight against malnutrition in all its manifestations [19]. In 2021, WHO and UNICEF collaborated to create an indicator for all children 0–23 months old, aimed at enhancing feeding practices and improving the health and development of young children [25]. This will hasten the process of combating child malnutrition.

Despite the widespread belief that complementary feeding should start at six months of age, research indicates that inappropriate timing of these practices continues to be a major global and national public health concern. In 2020, globally, one in four children (6–8 months old) did not receive any solid, semi-solid, or soft food. This delayed initiation of complementary feeding, which has been related to 45% of pediatric deaths, can lead to illness in children as well as inadequate growth and development [1, 2, 25, 26]. Ethiopia is among the developing nations with a high rate of infant death. According to recent predictions, the nation’s infant mortality rate is expected to be 43 deaths for every 1,000 live births, with an overall under-5 mortality rate of 55 deaths for every 1,000 live births. These figures show that the percentages of stunted, underweight, and wasted children under five are 37, 21, and 7%, respectively [18]. If all newborns received complementary feedings on time, all of these conditions would have decreased.

Previous studies has demonstrated that various factors, including sociodemographic, maternal, and obstetric-related variables, influence whether or not all mothers of any party initiate complementary feeding on time. However, previous studies has not adequately examined the effect of parity on time to initiate complementary feeding [18, 20–22, 27–34]. Despite the fact that many nations, including Ethiopia, create plans and implement WHO and UNICEF guidelines and indicators regarding the timely initiation of complementary feeding, the percentage of countries that do so remains low. However, research revealed that a disparity in parity plays a role in the general health of childrearing and development, including the use of appropriate complementary feeding practices, particularly for timely initiation, which was not noted in earlier studies. In Ethiopia, in particular, even though 62.5% of mothers start complementary feeding practices on time, a higher percentage of stunting remains a problem. This could be due to either 37.5% of mothers starting practices inappropriately, which is not examined by parity difference or other nutritionally related factors found by earlier studies [7–9, 18, 20–22, 25, 31, 32, 35, 36].

Besides, previous studies determined the timing of complementary feeding based on cross-sectional data, which did not show the temporal relationship in a better way, from all mothers without revealed the effect of parity. So that determining the effect of parity on the time to initiate complementary feeding using prospective cohort design and survival analysis modelling which would show the time in a better way than the tried past, and could generate new knowledge for scholars, healthcare administrators and policymakers to further develop a specific intervention. Therefore, this study will fill this gap by identifying the effect of parity on time to initiate complementary feeding and its confounders among mothers who gave live births in the Awi zone, Northwest Ethiopia.

## Methods and materials

### Study design and setting

A community-based prospective cohort study was carried out among primiparous and multiparous mothers who had a live birth in Awi Zone, Northwest Ethiopia. The study’s follow-up period was from December 1, 2022, to November 30, 2023. From December 1 to 30/2022 a baseline data collection was performed at both the Health facility and community level to recruit participants of the study; and each mother-infant pairs were followed from birth until initiation of complementary feeding or lost follow-up or 12 months age of the infant.

There are 1,342,324 people living in the Awi zone, and 684,590 of them are women. And 316,520 of the total population are women who are of reproductive age. According to data from the zonal health information system and a health department report for the second quarter of 2023, there were 41,746 under 12 months, 32,887 between the ages of 6 and 12 months and 8,913 under the age of 6 infants in the zone. The zone has one general hospital, 05 primary Hospitals, 49 Health centers, and 234 Health posts that gave health services to the zonal population.

### Populations, sample size and sampling procedures of the study

All mothers who had live births in Awi Zone were the source population whereas all mothers who had a recent live birth in selected districts in Awi Zone were the study population. Mothers who initiated complementary feeding at birth due to medical and cultural reasons were excluded from this study. Mothers were recruited based on the parity status, those having more than one live birth were multipara mothers (except twin birth in one labor) and those only having one live birth (including twin birth in one labor) were primipara mothers.

Using the general formula of time to event data in Stata version-17, the sample size was calculated under the following assumptions: 95% confidence level, 80% study power, population ratio of two comparison groups to one, the proportion of multiparous mothers who started complementary feeding on time was 47.3% (p1 = 0.473), assuming a 10% difference from multiparous mothers; therefore, the proportion of primipara mothers was 57.3% (p2 = 0.573), design effect 2, and 20% non-response rate [1]. Thus, 2146 mother-child pairs were the final estimated minimum adequate sample size. However, we used all 2395 mother-infant pairs that were found in the selected districts, in order to increase the study’s power and the findings’ representativeness to the zone’s source population. Thus, the sample size for this study involved 1576 multipara mothers and 819 primipara mothers.

All mothers who had live births in the selected four rural districts; Dangilla zuria (29 kebeles), Ayehu Guagussa (18 kebeles), Banja (22 kebeles) and Ankisha Guagussa (18 kebeles) were included in the study using simple random sampling techniques after 40% of the 9 rural districts of the zone were selected. All kebeles in the selected districts were included for the study. Initially, a preliminary survey was conducted to identify the total number of mothers who had live births within a month, and a 1: 2 ratio was used for the allocation of mothers into primiparity (Exposed group) and multi-parity (non-exposed group). Census was used as a sampling technique at selected districts to obtain a representative data for Awi zone. And 819 primi-para, and 1576 multi-para mothers were obtained at selected districts during the survey and a baseline interview was conducted to identify mothers eligible for the follow-up study. However, 87 primi-para and 112 multi-para mothers were excluded from the study due to medical and cultural reasons they were initiated complementary feeding at birth. Finally, a total of 2196 mothers, 732 primipara, and 1464 multipara mothers were included in the follow-up study.

### Variables

#### Dependent variable

Time to initiate complementary feeding.

#### Exposure variable

Parity.

#### Confounding variables

Age of the mother, educational status of the mother, husbands educational status, wealth status of the mother, religion, marital status of the mother, family size, sex of the child, media exposure, birth preparedness, birth plan, HIV status, birth type, Antenatal care, Postnatal care, place of delivery, and mode of delivery.

### Measurements

#### Outcome measurement

the time to initiate complementary feeding was measured from birth up to initiation of foods in addition to breast milk. Hence it is a prospective study, mother infant pairs were followed from birth until the start of complementary feeding or end of the study. Mothers not initiated complementary feeding for the infants or left out of the study was coded as “0” but initiated complementary feeding during the follow up period was coded as “1”, which was the event for this study.

### Exposure measurement

Parity was the exposure variable for this study. It was measured as primipara (coded as “1”), which was the exposure variable, and as Multipara (coded as “0”), which was the non-exposure category of the variable collected at baseline interview at the community and health facility level.

### Confounder measurement

Confounders, which were expected to be associated with both the parity and time to initiate complementary feeding were measured as follows. Age of the mother (“0” 15–24, “1” 25–34 “2” >=35), Educational status of the mother (“0” Normal education, “1” Primary education and “2” Secondary and above), husband educational status (“0” Normal education, “1” Primary education and “2” Secondary and above), wealth status (“0” Poorest, “1” poor “2” middle, “3” rich, “4” richest), religion (“0”orthodox, “1” Muslim and “2” other), marital status of the mother (“0” Unmarried, “1” married), family size (“0” <4 “1” ≥4), sex of the child (“0” male “1” Female), birth type (“0”Single “1” Twin), ANC (“0” No “1” 1–3 “2” ≥4), PNC (“0” No “1” Yes), place of delivery (“0” Home “1” Health Institution), birth plan (“0” No “1” Yes), and mode of delivery (“0” Vaginal “1” Cesarean Section).

**Birth preparedness**—if the mother practiced at least three of the five components of birth preparedness questions [37, 38], then considered as “Yes” otherwise “No”.

**Mass media exposure**—if the household has radio or television and listen at least once per week [18] was considered as having “Yes” and otherwise “No”.

**Incidence**—the occurrence of early, on and late initiation of complementary feeding during the follow up period and calculated as event per total mother-infant pairs-months.

### Data collection tools, procedures, and quality control

Questionnaires used for collecting data on exposure and confounding variables were adapted from previous published articles. Kobo collect v 2023.1.2 (https://www.kobotoolbox.org/) was used for data collection. Data were collected at the start of the follow up period; and on a monthly bases until the end of the follow up time. Thirty data collectors (B.Sc. health professionals) and 10 supervisors (Masters Health professionals), who have experienced in data collection and the culture of the community were participated in this study. Three days training was given for both of them. Besides, 30 community facilitators were involved in the data collection process. Data quality was ensured by translating the questionnaire from English to Amharic and then back to English to see consistency using assistance experts in both Amharic and English languages. Questionnaires was pre-tested on 5% of mother-infant pairs (36 primipara and 73 multipara mothers) from none selected districts within the zone. The principal investigator and supervisors conducted day-to-day follow-ups during the whole period of data collection. Each questionnaire was reviewed and checked for completeness by the supervisors and the principal investigators on each day as soon as possible and the necessary feedback was given to data collectors before leaving the data collection site. The principal investigator and co-investigators managed the overall activity of the study.

### Data management and analysis

Before the data was transferred to Stata 17 for more analysis, it was checked for consistency and completeness using the Kobo tool box and coded. Look for any missing data and determine if there were any notable outliers. Descriptive statistics, such as mean (standard deviation) for normally distributed continuous data and median (interquartile range), frequencies, and proportions for categorical data, were used to characterize the cohort’s features. Expert opinions and evidence from the literature were taken into consideration while choosing the confounders. The variance inflation factor (VIF) was used to evaluate the multicollinearity between the independent variables. The greatest VIF value was 1.40, and the mean VIF was 1.13 indicating that there was no multicollinearity issue.

**Fig. 1:**
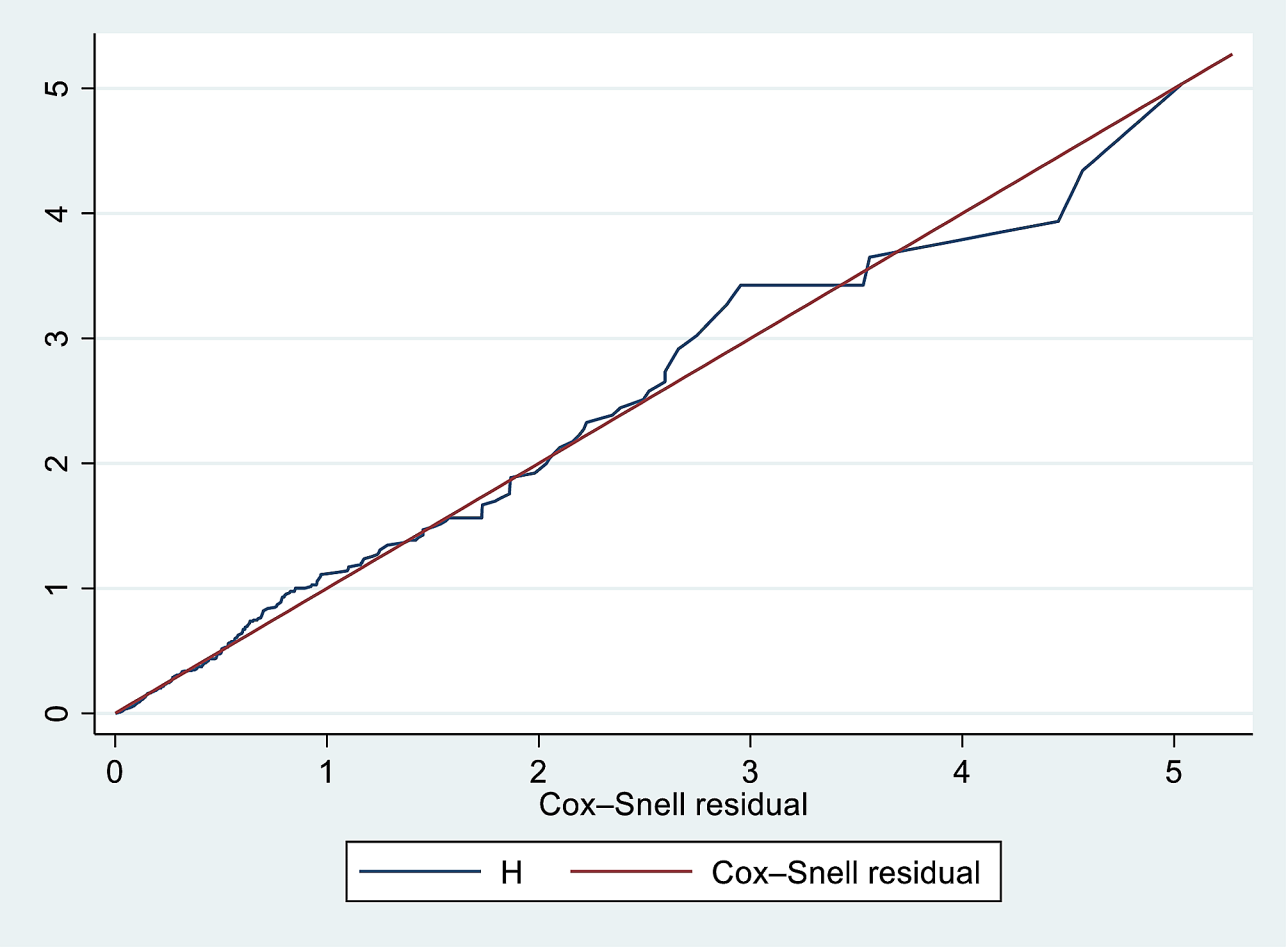
Cox-Snell residual curve for the effect of parity on time to initiate complementary feeding among Mother-infant pairs in Awi Zone, Northwest Ethiopia, 2023 (*N* = 2196)

The infants’ survival experience of exclusive breastfeeding at specific times between primipara and multipara mothers (Exposure variable) was assessed using the Kaplan-Meier survival curve and the log-rank test, as well as across a variety of categorical confounding variables. The statistical test of Schoenfeld residuals, and evidence of a time-dependent covariate (all variables had p-value > 0.05) was done. The fitness of the model was further assessed using the Cox-Snell residuals curve (Fig. 1) and the Schoenfeld residuals global test (P-value = 0.4861); tied failure observations were handled using the Breslow approach. In order to identify potentially relevant statistically significant (*P* ≤ 0.25) confounding variables in the bi-variable cox regression that would be taken into consideration in a multivariable Cox proportional hazards regression model. The multivariable analysis was performed using a model that included each of the selected variables. The number of events during the person-months of follow-up was used to compute the early or late incidence rate of complementary feeding initiation, and the median time to start it was finally established. The final model’s outcome was evaluated and expressed as a hazard ratio (HR) with 95% confidence intervals (CI). A p-value of less than 0.05 was used to declare statistical significance; and tables, graphs, or text narrations were used to present the findings.

## Results

A response rate of 91.69% was obtained from the 2395 mother-infant pairs who were enrolled in this prospective study, of which 2196 mothers were actually tracked until the mothers started complementary feeding or the follow-up study ended. Of these 8.31% of mothers, 10.62% and 7.12% were primipara and multipara mothers, respectively, who started complementary feeding at birth. Of the primipara mothers, 7.43% did so for cultural reasons (giving butter to the newborn), and 3.19% did so because of a breast problem. In addition, 7.12% of multipara mothers started complementary feeding because of cultural norms.

### Socio-demographic characteristics of the cohort

The median ± interquartile range (IQR) age of the mothers was 24 ± 7 (IQR = 21, 28) years with the minimum and maximum age of 15 and 46 years respectively. The median ± interquartile range age of the infants was 9 ± 3 (IQR = 8, 11) months with the minimum and maximum age of 6 and 12 months at the end of the follow up period respectively. Age of multiparous mothers was 24 ± 8 (IQR = 21, 29) years with the minimum and maximum age of 15 and 46 years respectively. Whereas the median ± IQR age of primiparous mothers was 24 ± 7 (IQR = 21, 28) years with the minimum and maximum age of 15 and 43 years respectively. Most of the mothers participated in this study had no formally educated (73.36%, unable to read and write (31.56%), able to read and write (41.80%)). In addition, the median ± IQR family size was 2 ± 1 (IQR = 2, 3) families with the minimum and maximum of 1 and 8 family members respectively (Table 1).

**Table 1:** Sociodemographic characteristics of the cohort for the effect of parity on time to initiate complementary feeding among mother-infant pairs in Awi Zone, Northwest Ethiopia, 2023 (*N* = 2196)

Variable name	Category	Mothers exposure status				Total (%)	X^2^ P-value
		Primipara (*N* = 732)		Multipara (*N* = 1464)			
		Event (%)	Censored (%)	Event (%)	Censored (%)		
Age of the mother	15–24	336 (85.71)	56 (14.29)	579 (78.03)	163 (21.97)	1,134 (51.64)	≤ 0.001+
	25–34	261 (84.47)	48 (15.53)	444 (72.55)	168 (27.45)	921 (41.94)	
	≥ 35	24 (77.42)	7 (22.58)	69 (62.73)	41 (37.27)	141 (6.42)	
Marital status of the mother	Single	38 (79.17)	10 (20.83)	148 (78.31)	41 (21.69)	237 (10.79)	0.462
	Married	577 (85.23)	100 (14.77)	914 (73.71)	326 (26.29)	1,917 (87.30)	
	Divorced	6 (85.71)	1 (14.29)	30 (85.71)	5 (14.29)	42(1.91)	
Education status of the mother	No education	436 (83.21)	88 (16.79)	806 (74.15)	281 (25.85)	1,611 (73.36)	0.090
	Primary education	95 (90.48)	10 (9.52)	151 (78.65)	41 (21.35)	297 (13.52)	
	Secondary and above	90 (87.38)	13 (12.62)	135 (72.97)	50 (27.03)	288 (13.11)	
Husband’s educational status	No education	333 (85.60)	56 (14.40)	558 (73.71)	199 (26.29)	1,146 (59.78)	0.736
	Primary education	171 (83.01)	35 (16.99)	231 (73.10)	85 (26.90)	522 (27.23)	
	Secondary and above	73 (89.02)	9 (10.98)	125 (74.85)	42 (25.15)	249 (12.99)	
Sex of the infant	Male	262 (85.62)	44 (14.38)	503 (75.53)	163 (24.47)	972 (44.26)	0.481
	Female	359 (84.27)	67 (15.73)	589 (73.81)	209 (26.19)	1,224 (55.74)	
Religion	Orthodox	612 (84.88)	109 (15.12)	1,080(74.53)	369 (25.47)	2,170 (98.82)	0.732
	Muslim	9 (81.82)	2 (18.18)	12 (80.00)	3 (20.00)	26 (1.18)	
Family size	< 4	570 (85.59)	96 (14.41)	876 (74.87)	294 (25.13)	1,836 (83.61)	0.054
	≥ 4	51 (77.27)	15 (22.73)	216 (73.47)	78 (26.53)	360 (16.39)	
Wealth status	Poorest	110 (85.94)	18 (14.06)	246 (76.64)	75 (23.36)	449 (20.45)	0.052
	Poor	125 (80.65)	30 (19.35)	177 (68.60)	81 (31.40)	413 (18.81)	
	Middle	132 (87.42)	19 (12.58)	187 (70.57)	78 (29.43)	416 (18.94)	
	Rich	129 (84.31)	24 (15.69)	220 (76.66)	67 (23.34)	440 (20.04)	
	Richest	125 (86.21)	20 (13.79)	262 (78.68)	71 (21.32)	478 (21.77)	
Age of the infant (end of follow up)	6–8 months	237 (82.58)	50 (17.42)	423 (74.87)	142 (25.13)	852 (38.80)	0.626
	9–12 months	384 (86.29)	61 (13.71)	669 (74.42)	230 (25.58)	1,344 (61.20)	
Media Exposure	Yes	300 (86.46)	47 (13.54)	505 (75.60)	163 (24.40)	1,015 (46.22)	0.171
	No	321 (83.38)	64 (16.62)	587 (73.74)	209 (26.26)	1,181 (53.78)	

+ - statistical association present

### Obstetric and maternal characteristics of the cohort

The mean ± standard deviation (SD) of antenatal care services for all parity mothers was 3.43 ± 1.52. The mean ± standard deviation (SD) of antenatal care services among primipara mothers was 3.38 ± 1.43. The mean ± (SD) of antenatal care services among multipara mothers was 3.46 ± 1.56. Around 84.84% of primipara and 74.59% of multipara mothers initiated complementary feeding for their infants during the follow up period. But a total of 483 (21.99%) of mothers were not initiated complementary feeding for their infants until the end of the study period (Table 2).

**Table 2:** Obstetric and maternal characteristics for the effect of parity on time to initiate complementary feeding among mother-infant pairs in Awi Zone, Northwest Ethiopia, 2023 (*N* = 2196)

Variable name	Category	Mothers exposure status				Total (%)	X^2^ P-value
		Primipara (*N* = 732)		Multipara (*N* = 1464)			
		Event (%)	Censored (%)	Event (%)	Censored (%)		
Parity	Primipara	Not applicable				732 (33.33)	< 0.001+
	Multipara					1,464 (66.67)	
Antenatal care	No	25 (78.13)	7 (21.88)	56 (74.67)	19 (25.33)	107 (4.87)	0.624
	≤ 3	283 (85.24)	49 (14.76)	480 (73.39)	174 (26.61)	986 (44.90)	
	≥ 4	313 (85.05)	55 (14.95)	556 (75.65)	179 (24.35)	1,103 (50.23)	
Birth preparedness	Yes	529 (85.74)	88 (14.26)	911 (74.79)	307 (25.21)	1,835 (83.56)	0.232
	No	92 (80.00)	23 (20.00)	181 (73.58)	65 (26.42)	361 (16.44)	
Birth plan	Yes	470 (85.92)	77 (14.08)	759 (74.70)	257 (25.30)	1,563 (71.17)	0.266
	No	151 (81.62)	34 (18.38)	333 (74.33)	115 (25.67)	633 (28.83)	
Place of Birth	Home	85 (81.73)	19 (18.27)	201 (71.79)	79 (28.21)	384 (17.49)	0.066
	Health Institution	536 (85.35)	92 (14.65)	891 (75.25)	293 (24.75)	1,812 (82.51)	
Birth type	Single	598 (85.19)	104 (14.81)	1,043(74.0)	366 (25.98)	2,111 (96.13)	0.049+
	Twin	23 (76.67)	7 (23.33)	49 (89.09)	6 (10.91)	85 (3.87)	
Mode of delivery	SVD*	515 (84.70)	93 (15.30)	869 (73.64)	311 (26.36)	1,788 (81.42)	0.155
	CS**	106 (85.48)	18 (14.52)	223 (78.52)	61 (21.48)	408 (18.58)	
Postnatal care	Yes	521 (85.41)	89 (14.59)	877 (75.28)	288 (24.72)	1,775 (80.83)	0.079
	No	100 (81.97)	22 (18.03)	215 (71.91)	84 (28.09)	421 (19.17)	
HIV test	Yes***	572 (85.63)	96 (14.37)	985 (74.79)	332 (25.21)	1,985 (90.39)	0.133
	No	49 (76.56)	15 (23.44)	107 (72.79)	40 (27.21)	211 (9.61)	

* spontaneous vaginal delivery, ** Cesarean section *** all negative (there may have bias)

### Exclusive breastfeeding survival status

**Fig. 2:**
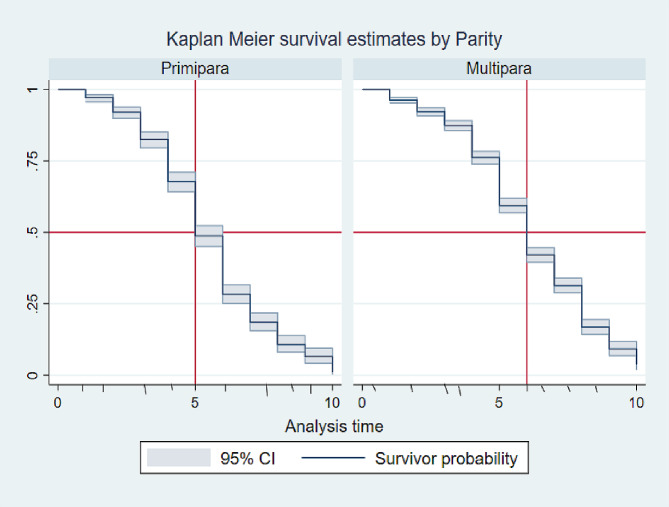
The overall Kaplan Meier survival estimates of the effect of parity on time to initiate complementary feeding among mother-infant pairs in Awi Zone, Northwest Ethiopia, 2023 (*N* = 2196)

**Fig. 3:**
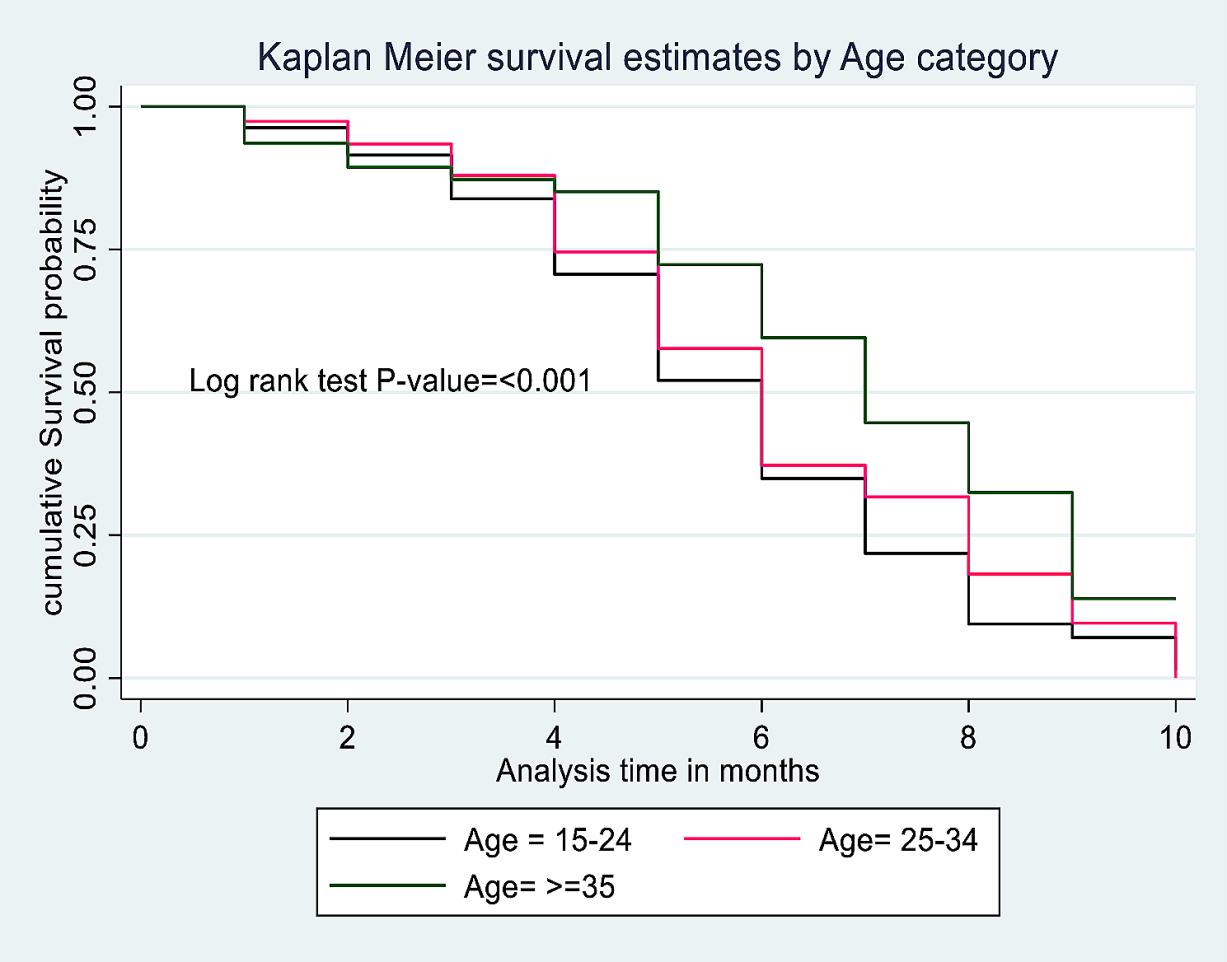
Kaplan Meier survival estimates by age category for the effect of parity on time to initiate complementary feeding among mother-infant pairs in Awi Zone, Northwest Ethiopia, 2023 (*N* = 2196)

**Fig. 4:**
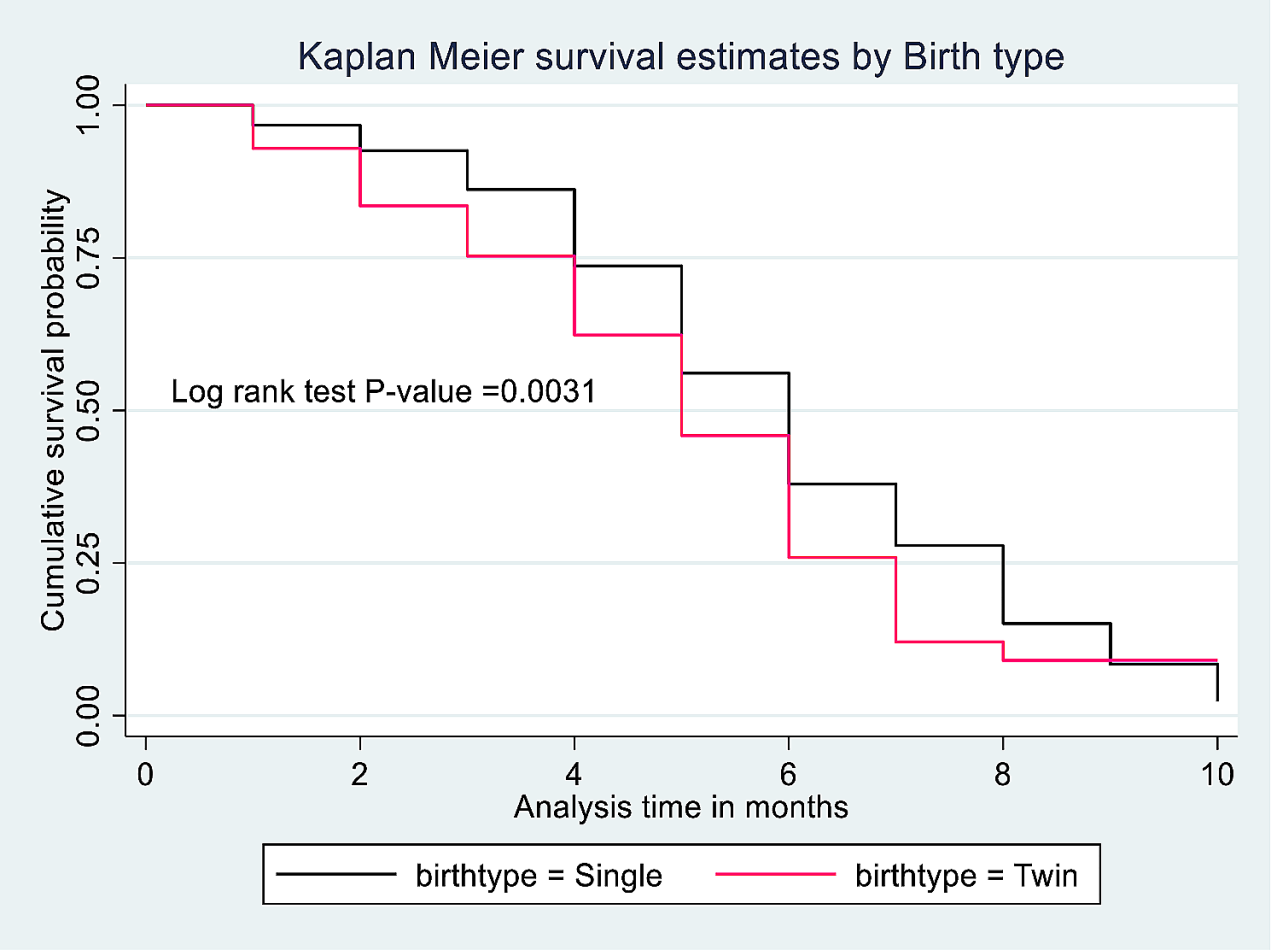
Kaplan Meier survival estimates by birth type for the effect of parity on time to initiate complementary feeding among mother-infant pairs in Awi Zone, Northwest Ethiopia, 2023 (*N* = 2196)

From the total of mothers participated in this study, 483 (21.99%) were survived on exclusive breastfeeding for their infants until the end the follow up period with the median ± IQR survival time of 6 ± 4 months (IQR = 4, 8). Of them, 372 (16.94%) were multipara mothers with the median ± IQR survival time of 6 ± 3 months (IQR = 5, 8) whereas the median ± IQR survival time of primipara mothers was 5 ± 3 months (IQR = 4, 7) (Fig. 2). Kaplan Meier survival estimates for statistically significant predictors were showed in (Figs. 3 and 4).

The median ± IQR time of initiating complementary feeding of all parity mothers for their infants was 6 ± 3 (IQR: 4, 7), 6 ± 4 (IQR:4, 8)and 7 ± 4 (IQR:5, 9) months by the age category of 15–24, 25–34 and ≥ 35 years respectively. The median ± IQR time of initiating complementary feeding of primipara mothers for their infants was 5 ± 3 (IQR: 4, 7), 6 ± 3 (IQR: 4, 7) and 7 ± 2 (IQR: 5, 7) months by the age category of 15–24, 25–34 and ≥ 35 years respectively. Besides, The median ± IQR time of initiating complementary feeding of multipara mothers for their infants was 6 ± 3 (IQR: 4, 7), 6 ± 3 (IQR:5, 8)and 8 ± 4 (IQR:5, 9) months by the age category of 15–24, 25–34 and ≥ 35 years respectively.

Similarly, The median ± IQR time of initiating complementary feeding of all parity mothers who had a singleton infant was 6 ± 4 (IQR: 4, 8) months, whereas who had twin infants was 5 ± 3 (IQR:4, 7) months. The median ± IQR time of initiating complementary feeding of primipara mothers who had a singleton infant was 5 ± 3 (IQR: 4, 7) months, whereas who had twin infants was 6 ± 3 (IQR:4, 7) months. Besides, The median ± IQR time of initiating complementary feeding of multipara mothers who had a singleton infant was 6 ± 3 (IQR: 5, 8) months, whereas who had twin infants was 5 ± 3 (IQR:3, 6) months.

### Incidence of complementary feeding initiation

Out of these initiated complementary for their infants until the end of the follow-up period, 972 (44.26%), 401 (18.26%) and 340 (15.48%) initiated early, on time and late to 6 months. Out of them initiated early, on time and lately, 596 (27.14%), 252 (11.48%) and 244 (11.11%) were multipara mothers respectively.

The overall incidence rate of complementary feeding initiation within the follow-up period was 14.25 (95%CI: 13.59, 14.94) per 100 person month observations after 12,024 risk follow up months. The early, on time and late incidence of complementary feeding initiation were 9.88 (95%CI: 9.28, 10.53), 32.76 (95%CI: 29.71, 36.13) and 35.19 (95%CI: 31.65, 39.14) per 100 person months observations after 9834, 1800 and 340 risk follow up months respectively. Whereas the incidence rate of complementary feeding initiation within the follow-up period among multipara mothers was 13.30 (95%CI: 12.53, 14.12) person months observation after 8208 risk follow up months. In addition, 9.01 (95%CI: 8.31, 9.76), 29.03 (95%CI: 25.66, 32.85) and 33.79 (95%CI: 29.81, 38.31) per 100 person months’ observation was occurred at early, on time and lately to the 6 months among the multipara mothers after 6618, 868 and 722 risk follow up months respectively. The overall incidence rate of initiation of complementary feeding among primipara mothers was 16.27 (95%CI: 15.04, 17.61) person months’ observations after 3816 risk follow up months. The early, on time and lately incidence rate of initiation of complementary feeding among primipara mothers were 11.69 (95%CI: 10.57, 12.94), 41.85 (95%CI: 35.39, 48.84) and 39.75 (95%CI: 32.58, 48.51) person months observation after 3216, 356 and 244 risk follow up months respectively.

### Predictors of time to complementary feeding initiation

After the confounders were adjusted, the main exposure variable (Parity) in this study was identified as a statistically significant predictor for time to initiate complementary feeding. Primipara mothers had a 30% higher rate to initiate complementary feeding than multipara mothers (AHR = 1.30, 95%CI: 1.17, 1.43; P-value < 0.001) (Table 3).

Among the confounders considered in this study, age of the mother and birth type were identified as a statistically significant predictors for time to initiate complementary feeding. Age from 15 to 24 and 25–34 were higher rate of complementary feeding initiation than age ≥ 35 years (AHR = 1.69, 95%CI: 1.36, 2.09; P-value < 0.001 and AHR = 1.45, 95%CI: 1.17, 1.81; P-value = 0.001). Initiating complementary feeding was 1.29 times higher rate among mothers gave twin birth than mothers gave single birth (AHR = 1.29, 95%CI: 1.02, 1.64; P-value = 0.028) (Table 3).

**Table 3:** Predictors of time to initiate complementary among mother-infant pairs in Awi Zone, Northwest Ethiopia, 2023 (*N* = 2196)

Variable		Complementary feeding initiation status		CHR (95%CI)	AHR (95%CI)	X^2^ p-value
		Event	Censored			
Parity	Primipara	621	111	1.32 (1.19, 1.46)	1.30 (1.17, 1.43)**	< 0.001
	Multipara	1,092	372	1	1	
Sex	Male	768	204	1.02 (0.93, 1.13)	1.03 (0.93, 1.14)	0.310
	Female	945	279	1	1	
Age	15–24	915	219	1.74 (1.40, 2.15)	1.69 (1.36, 2.09)**	< 0.001
	25–34	705	216	1.48 (1.19, 1.84)	1.45 (1.17, 1.81)**	
	≥ 35	93	48	1	1	
Family size	< 4	1,446	390	1	1	0.054
	≥ 4	267	93	0.95 (0.84, 1.09)	1.01 (0.88, 1.16)	
Mass media exposure	Yes	804	211	1.08 (0.98, 1.18)	1.06 (0.96, 1.17)	0.206
	No	909	272	1	1	
Wealth index	Poorest	358	91	1	1	0.035
	Poor	301	112	1.01 (0.86, 1.17)	0.94 (0.80, 1.10)	
	middle	318	98	0.98 (0.85, 1.15)	0.93 (0.79, 1.08)	
	Rich	349	91	1.07 (0.93, 1.24)	1.03 (0.88, 1.20)	
	Richest	387	91	1.16 (1.01, 1.35)	1.11 (0.95, 1.29)	
Antenatal care	No	81	26	0.90 (0.71, 1.13)	1.03 (0.79, 1.36)	0.571
	< 4	762	224	1.01 (0.92, 1.12)	1.03 (0.93, 1.14)	
	≥ 4	870	233	1	1	
Birth preparedness	Yes	1,440	395	1	1	0.232
	No	273	88	0.91 (0.80, 1.03)	0.90 (0.77, 1.06)	
Place of birth	Home	286	98	1	1	0.066
	Health institution	1,427	385	1.15 (1.02, 1.31)	0.92 (0.68, 1.27)	
Birth type	Singleton	1,641	470	1	1	0.028
	Twin	72	13	1.37 (1.08, 1.73)	1.29 (1.02, 1.64)*	
Postnatal care	Yes	1,398	377	1	1	0.079
	No	315	106	0.86 (0.76, 0.97)	0.84 (0.62, 1.14)	

## Discussion

In this study, parity was identified as having a statistically significant effect for time to initiate complementary feeding. Being primipara mother was a higher rate to initiate complementary feeding early compared to multipara mothers (51.37% and 40.71% respectively). The findings from Northern Ethiopian study showed that mothers having two and more child introduce complementary feeding based on the recommendation, which is consistent with the present study’s finding which stated that 11.48% multipara mothers initiated complementary feeding on time than 6.79% of primipara mothers [39]. It may be justified as: the mothers’ level of understanding about infants’ nutritional practice will increase while their parity increases. In addition primipara mothers are less confident on their breast milk adequacy to the infant even though none of previous literatures explored. Due to this, primipara mothers initiating additional foods early to the infant by assuming additional foods give better nutritional value than breastfeeding only. Multipara mothers may have more exposures for health education about breastfeeding and complementary feeding counselling in previous pregnancies and child caring practices. This may have an impact for subsequent child caring practices. Whereas, this finding is inconsistent with the findings of the study done in Brazil, which revealed that parity has no effect on the initiation of complementary feeding [40]. Another study in Brazil revealed that Multiparity has a protective effect for early initiation of complementary feeding [32]. The reason behind may be the present study includes mothers gave birth from both at health facility and community level in opposite to the Brazilian study which included only mothers gave birth at Hospital level. Our study further revealed that multipara mothers lately initiated complementary feeding to the infants than primipara mothers (11.11% and 4.37% respectively). Because of this, the median time to initiate complementary feeding was earlier among primipara mothers (5 months) than multipara mothers (6 months). It may be due to- the present study found that primipara mothers got less antenatal care services than multipara mothers. This further explained that primipara mothers got less breastfeeding and complementary feeding counselling services than multipara mothers.

The incidence rate of early and late initiation of complementary feeding among primipara mothers was higher than multipara mothers. It is in line with the findings of the study done in Ethiopia, which revealed that primipara mothers initiated complementary feeding inappropriately (either early or lately) than multipara mothers [41]. This may be the difference in child caring and feeding experience between multi and primipara mothers. Besides, the knowledge and attitude of primipara mothers may be different in child feeding practice than multipara mothers. In the same way, the findings of the study in two European countries shown that multiple parity has a factor for initiation of complementary feeding after 6 completed months than primipara mothers in Australia [42]. Which implied that primipara mothers initiated complementary feeding early than multipara mothers. Whereas our study finding was in contrast to the Netherland study’s finding, which explained that multipara mothers introduce complementary feeding between 3 and 6 months than primipara mothers [43]. The reason for the difference may be the sampling technique used by our study was probability sampling in opposite to the Netherland study, which was used a parent driven sampling procedure where it is a non-probability sampling technique. This may affect the overall incidence and prevalence of early and late initiation of complementary feeding.

In addition to the main exposure variable, being a younger age (15–24 and 25–34 years) was a statistically significant risk factor for early or late initiation of complementary feeding than older age mothers (≥ 35 years). It is consistent with the findings from two European countries study, which explained that lower maternal age is associated with early introduction of complementary feeding [42]. This might be associated with the difference in the knowledge and attitude of adolescent mothers and adult mothers regarding about timely initiation of complementary feeding. In addition to that mothers born their first baby lately care more for their infants than mothers born their babies at an early age due to the Ethiopian cultural value given. In the same way, the findings of the New Zealand study revealed that younger age of the mother (< 30 years old) was a significant factor for early introduction of complementary feeding than the older mothers [44]. However, a study in Northern Ethiopia showed that age of the mother had no statistical association with the timely introduction of complementary feeding [23]. The difference may be associated with the study settings, where our study was conducted at community level by including both home and health institution delivered mothers in opposite to the Northern Ethiopian study, which included mothers infant pairs come to health facility for under-five health care visits [23].

Besides, a mother who born twin has a higher risk to initiate complementary feeding than the mothers who born single. Twin born mothers may face difficulty to sufficiently breastfeed the infants. So that the mothers introduce complementary feeding early. Particularly primipara mothers (Initiated complementary feeding more earlier than multipara mothers in this study) have a lower level of confidence on their breast milk sufficiency than multipara mothers [45, 46]. These two factors together increased the early initiation of complementary feeding in opposite to the WHO and UNICEF recommendation [5, 26, 47]. However, the study done in France stated that type of birth (Singleton/ Twin) had no a statistically significant factor for time to complementary feeding initiation [48]. This opposite finding may be due the population recruitment technique difference for the study. The present study recruits mothers from the community for this research purpose only but the France study analyzed the data from longitudinal birth cohort collected for different nationally representative purposes [49].

This study has faced limitations. Due to inaccessibility or unavailability of previous studies on the incidence of early and late initiation of complementary feeding by parity consideration, we discussed the findings of this study by approximation to the findings of the previous studies.

### Conclusion and recommendation

Parity was identified as a statistically significant predictor for time to initiate complementary feeding. The incidence rate of early and late initiation of complementary feeding was higher among primipara mothers than multipara mothers. Besides, the median time to initiate complementary feeding was lower among primipara mothers than multipara mothers. So, a parity based complementary feeding practice education should be advocated to tackle the gap and further reduce infants and children malnutrition prevalence. Relatively younger age and twin delivered mothers could initiate complementary feeding against the recommendation. Therefore, intervention considering such statistically significant predictors could have a public health importance.

### Electronic supplementary material

Below is the link to the electronic supplementary material.

Supplementary Material 1

Supplementary Material 2

Supplementary Material 3

## Data Availability

All data are included with in the manuscript.
