# Supplementary material for: The effect of parity on time to initiate complementary feeding among mother-infant pairs in Awi Zone, Northwest Ethiopia
Source: Ital J Pediatr. 2024 Mar 13;50:49. doi: 10.1186/s13052-024-01612-1 (PMC10936086; doi:10.1186/s13052-024-01612-1)
Supplement: Supplementary file 2 — Supplementary Material 2 [file 13052_2024_1612_MOESM2_ESM.docx]

# Annexs

## **Annex I: English version Information Sheet**

Greeting!

Dear respondent! My name is________ and I am working as a data collector for the study being conducted in this Zone by Injibara University College of Medicine and Health Sciences. You are selected to be one of the participants in this study and you are kindly requested to answer for listed questionnaires below.

**Study title: The effect of parity on** time to initiate complementary feeding among mother-infant pairs in Awi zone, Northwest Ethiopia, 2023

**Procedure and duration**: there are different questions to be answered during the interview. The interview process will take 10-15 minutes.

**Risks:** There is no risk associated with taking part in the study. Every piece of information will be kept private.

**Benefit:** no direct benefit of being involved in this study but the information you provide is very important to solve problems associated with the timely initiating of complementary feeding in prim parous women.

**Incentives**: There is no incentive or payment to be gained by taking part in this project.

**Confidentiality:** The information you provide us will be kept confidential. The questionnaire will be coded to exclude showing your name on the questionnaire and consent form.

**Rights:** Participation in this study is voluntary. You have the right to declare not to participate in this study and you have the right to withdraw from participating at any time.

**Contact address:** If there is any question or unclear idea at any time about the study or the procedures, do not hesitate to contact and speak to the principal investigator at the address shown below.

**Phone number:** +251920763630

**E-mail address:** mkdt2121@gmail.com

I have read this form and would you understand the conditions stated above? If so are you willing to participate in this study?

No (say thank you) 2. Yes (continue interviewing)

**Participant’s Signature ----------------------------- Date** ------------------------------------

**Interviewer’s name-----------------------Interviewer’s signature --------------- Date-----------------**

**Supervisor’s Name-------------------------Supervisor’s Signature---------------Date ----------------**

**Kebele of the participant _____________________________________**

**Part I: Sociodemographic predictors**

1. Age of mother at infant’s birth-----------------------
2. Marital status at infant’s birth
3. Single
4. Married
5. Divorced
6. Widowed
7. List if any……………….
8. Educational status of the mother at infant’s birth
9. Unable to read and write
10. Able to read and write
11. Elementary education
12. Secondary education
13. University education and above
14. List if any……………….
15. Educational status of the Husband/ spouse/ friend at infant’s birth
16. Unable to read and write
17. Able to read and write
18. Elementary education
19. Secondary education
20. University education and above
21. List if any……………….
22. Sex of the child 1. Male 2. Female
23. Ethnicity 1. Agew 2. Amhara 3. Oromo 4. Tigre 5. Gumuz 6. List if any………
24. Religion 1. Orthodox 2. Muslim 3. Protestant 4. Catholic 5. List if any……………
25. Family size (Excluding the current infant)__________________
26. Wealth Index tools------------------------------

1. Land 1. Yes 2. No

2. Hectare_____________

3. Maize 1. Yes 2. No

4. Barley/Wheat 1. Yes 2. No

5. Pea/ Bean 1. Yes 2. No

6. Teff 1. Yes 2. No

7. Radio ownership 1. Yes 2. No

8. Cow/ox 1. Yes 2. No

9. Horse/mule 1. Yes 2. No

10. Sheep/Goat 1.Yes 2. No

11. Cart 1. Yes 2. No

12. Town house 1. Yes 2. No

13. Beehive 1. Yes 2. No

14. Plough plow 1. Yes 2. No

15. Shovel 1. Yes 2. No

16. Sickle 1. Yes 2. No

17. Hoe 1. Yes 2. No

18. Axe 1. Yes 2. No

**Part II: Time to initiate complementary feeding measuring variables**

1. Date of birth of the infant? …………………………………………. (DD/MM/YY)
2. Age of the infant……………………………in days.
3. Have you born a baby before? 1. Yes 2. No
4. What is your parity status? 1. Primiparous 2. Multiparous
5. Did you initiate complementary feeding for your infant?
6. Yes
7. No
8. If yes to question 202, what is the date of initiate of complementary feeding …....................................................(DD/MM/YY)
9. How many days passed for this infant after the date of initiate of complementary feeding………………………………(DD/MM/YY)
10. Total duration on Exclusive breastfeeding……………………..in days.

**Part III: Maternal and Obstetric predictors**

1. Antenatal care follow up during her pregnancy
2. Yes
3. No
4. If yes to question 201, how many times did you visit……………..
5. Did you have any birth preparedness during your pregnancy for this infant? 1 Yes 2 No
6. Have you been planned to delivery at Health institution? 1. Yes 2. No
7. Have you been planned to ANC services at Health institution? 1. Yes 2. No
8. Have you been prepared for transport to Health institution if labor starts? 1. Yes 2. No
9. Have you been ready to accept blood if donation require? 1. Yes 2. No
10. Did you able to list three of pregnancy related danger signs?____________________________
11. Where did you born your infant?
12. Home
13. Hospital
14. Health center
15. Health Post
16. Private Hospital
17. Private clinic
18. By what mode did you born your infant?
19. Vaginally Normal
20. Vaginally instrumental
21. Cesarean section
22. How many neonates did you born at your labor?
23. Single
24. Twin
25. List if any…………………
26. Did you born this infant with your plan?
27. No
28. Yes
29. Did you follow at least the first 48 hours postnatal care visit?
30. Yes
31. No
32. Have you ever been tested for HIV?
33. Yes
34. No
35. If yes to Q 208, could you tell us your result please?
36. Positive
37. Negative

**Thank you for your participation**
