# Supplementary material for: The effect of parity on time to initiate complementary feeding among mother-infant pairs in Awi Zone, Northwest Ethiopia
Source: Ital J Pediatr. 2024 Mar 13;50:49. doi: 10.1186/s13052-024-01612-1 (PMC10936086; doi:10.1186/s13052-024-01612-1)
Supplement: Supplementary file 3 — Supplementary Material 3 [file 13052_2024_1612_MOESM3_ESM.docx]

Dear Italian Journal of pediatrics editorial manager,

We wish to submit a research paper entitled “The Effect of Parity on Time to Initiate Complementary Feeding among Mother-infant pairs in Awi Zone, Northwest Ethiopia” to the Italian journal of Pediatrics.

In this paper, we reported that the median time to complementary feeding initiation among 732 primipara and 1464 multipara mother-infant pairs 5 and 6 months respectively. This finding is important for decision-making because the more than the average number of primipara mothers initiated complementary feeding for the infants before 6 months of age of the infants in opposite to the WHO/UNICEF recommendation.

This study was designed to reduce potential confounding factors and biases. The data were statistically analyzed and confident that our conclusions are well-founded. We believe that the topic of our research falls within the scope of your Journal and that these findings will be of interest to its readers. Data collected during this study is available at the corresponding author.

Our paper has 7996 words and contains 4 figures and 3 tables. This study has not been published previously and is not under consideration elsewhere as much as we have searched. There is no funding agent/s for this paper. Authors have no conflicts of interest to disclose, and all approved this submission.

Thank you for your consideration.

Best regards,

Tilahun Degu Tsega

Lecturer in Public Health Epidemiology

Injibara University, College of Medicine and Health Sciences

[mkdt2121@gmail.com](mailto:mkdt2121@gmail.com)
